# Development and validation of prognosis model of mortality risk in patients with COVID-19

**DOI:** 10.1017/S0950268820001727

**Published:** 2020-08-04

**Authors:** Xuedi Ma, Michael Ng, Shuang Xu, Zhouming Xu, Hui Qiu, Yuwei Liu, Jiayou Lyu, Jiwen You, Peng Zhao, Shihao Wang, Yunfei Tang, Hao Cui, Changxiao Yu, Feng Wang, Fei Shao, Peng Sun, Ziren Tang

**Affiliations:** 1 AI Research Division, A.I. Phoenix Technology Co., Ltd, Hong Kong, China; 2 Research Division for Mathematical and Statistical Science, University of Hong Kong, Hong Kong, China; 3 Department of Emergency Medicine, Union Hospital, Tongji Medical College, Huazhong University of Science and Technology, Wuhan, China; 4 Department of Emergency Surgery, The west campus of Union Hospital, Tongji Medical College, Huazhong University of Science and Technology, Wuhan, China; 5 Department of Emergency Medicine, Beijing Chaoyang Hospital, Capital Medical University, Beijing, China; 6 Department of Respiratory and Critical Care Medicine, Beijing Chaoyang Hospital, Capital Medical University, Beijing, China; 7 Beijing Institute of Respiratory Medicine, Beijing Engineering Research Center for Diagnosis and Treatment of Respiratory and Critical Care Medicine, Beijing Chaoyang Hospital, Capital Medical University, Beijing, China; 8 Beijing Key Laboratory of Respiratory and Pulmonary Circulation Disorders, Beijing, China; 9 Beijing Key Laboratory of Cardiopulmonary Cerebral Resuscitation, Beijing, China

**Keywords:** COVID-19, machine-learning methods, mortality risk, prognosis, Random Forest

## Abstract

This study aimed to identify clinical features for prognosing mortality risk using machine-learning methods in patients with coronavirus disease 2019 (COVID-19). A retrospective study of the inpatients with COVID-19 admitted from 15 January to 15 March 2020 in Wuhan is reported. The data of symptoms, comorbidity, demographic, vital sign, CT scans results and laboratory test results on admission were collected. Machine-learning methods (Random Forest and XGboost) were used to rank clinical features for mortality risk. Multivariate logistic regression models were applied to identify clinical features with statistical significance. The predictors of mortality were lactate dehydrogenase (LDH), C-reactive protein (CRP) and age based on 500 bootstrapped samples. A multivariate logistic regression model was formed to predict mortality 292 in-sample patients with area under the receiver operating characteristics (AUROC) of 0.9521, which was better than CURB-65 (AUROC of 0.8501) and the machine-learning-based model (AUROC of 0.4530). An out-sample data set of 13 patients was further tested to show our model (AUROC of 0.6061) was also better than CURB-65 (AUROC of 0.4608) and the machine-learning-based model (AUROC of 0.2292). LDH, CRP and age can be used to identify severe patients with COVID-19 on hospital admission.

## Introduction

Since the outbreak of the novel coronavirus resulting in coronavirus disease 2019 (COVID-19) in Wuhan, China, at the end of 2019, there have been more than 12.5 million individuals in more than 200 countries with confirmed COVID-19, of whom more than 560 000 have died as on 13 July 2020 [1]. The type of pneumonia caused by COVID-19 is highly infectious, and the World Health Organization (WHO) has declared the ongoing outbreak as a pandemic with high short-term mortality rate [1]. Several studies have already been reported regarding the clinical course and outcomes of patients with COVID-19 pneumonia [2–6]. The mortality of patients and risk factors of prognosis were studied [2, 3, 6–15]. The prognosis of patients with COVID-19, and the identification of clinical variables of mortality risk after hospital admission are still challenging problems [6–15]. Several prediction methods [7–15] were studied to guide clinical decisions. However, their analysed data sets may be limited, their proposed methods may be poorly reported, or their reported performance may be optimistic [16].

In this study, we aimed to investigate machine-learning methods to rank clinical features, and multivariate logistic regression method to identify clinical features with statistical significance in prediction of mortality risk in patients with COVID-19 using their clinical data on admission at the west campus of Union Hospital in Wuhan.

## Methods

### Study design

This was a single-centred, retrospective, observational study. We considered the medical information of inpatients with COVID-19 collected between 15 January and 15 March 2020. The eligibility criteria were as follows: patients aged 14 years or older and patients who were diagnosed with COVID-19 pneumonia according to the interim guidelines from the World Health Organization. The patients were labelled as survivor or non-survivor. The study was approved by the Ethics Committee Board of Beijing Chaoyang Hospital, Capital Medical University and Union Hospital, Tongji Medical College, Huazhong University of Science and Technology, and the requirement for informed consent was waived.

### Data collection

Symptoms, comorbidity, demographic, vital signs, CT scans and laboratory were extracted from electronic medical records of inpatients with COVID-19 in Union Hospital by using a standardised data collection form. For all the patients included in the data set, we only included patients with laboratory results within a 24-h admission period.

We identified in-sample data of patients who had symptoms, comorbidity, demographic, vital sign, CT scans and laboratory on admission without missing values in the study. In addition to the in-sample data set, we further collected samples with COVID-19 for out-sample data. Note that out-sample patients had some missing values in the other clinical measurement variables, they were not selected in the in-sample data set.

### Statistical methods

On the basis of the in-sample data set, we implemented a series of data analysing techniques and found the top laboratory features highly related to the mortality rate of a patient. To test the stability of our model, we made a comparison test with other models and the out-sample data.

In this study, we employed supervised Random Forest [17] and XGBoost [18] classifiers as the predictor models for ranking variables. Both Random Forest and XGBoost are machine-learning algorithms based on tree-based classification models. Random Forest utilises bagging in training, while XGBoost makes use of re-labels in training. However, their black-box models are difficult to interpret the mortality risk in patients. Here we only used them to calculate the relative importance of each variable in the discriminative model for the two labels survivor and non-survivor of in-sample patients.

In the next step, we used multivariate logistic regression [19] to identify variables by checking their statistical significance from the list of selected variables in the output by the Random Forest and XGBoost. Validation was assessed by the *z*-score of each variable based on 500 bootstrapped samples. A *P*-value <0.05 was considered statistically significant. The resulting multivariate logistic regression was then constructed based on these statistically significant variables of the in-sample patients to determine the mortality prediction model. Here four-fold cross-validation (75% training and 25% testing data of in-sample patients) is employed to obtain the resulting mortality model. The CURB-65 [7] method and the machine-learning-based model on XGBoost [13] were also compared with the obtained mortality prediction model. In addition to the in-sample data set, an out-sample data set of out-sample patients were collected and used to predict mortality by the determined prediction model, then compared with the CURB-65 method and the machine-learning-based model on XGBoost.

We used area under the receiver operating characteristics (AUROC) as the precision measurement. By comparing the true-positive and false-positive numbers, a receiver operating characteristics (ROC) curve is a graph showing the performance of a classification model at all classification thresholds. AUROC measures the entire two-dimensional area underneath the entire ROC curve. It tells how much model is capable of distinguishing between classes. Higher the AUROC, better will be the model at predicting different classes, for example, distinguishing patients with disease and no disease.

## Results

**Fig. 1.**
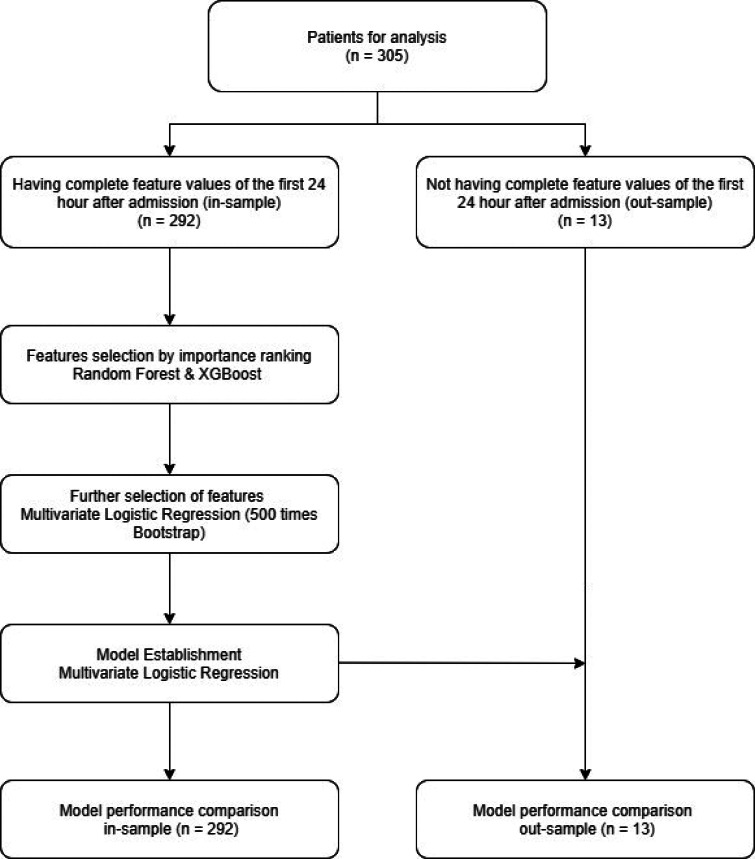
Flow chart of the study process.

We collected data from 305 patients (292 in-sample data and 13 out-sample data) with 33 variables each and the baseline characteristics of patients are shown in Table 1. In-sample patients' data were further labelled as survivor and non-survivor. They included 57 non-survivor patients (19.5%) and 235 survivor patients (79.5%). The study process is shown in the form of a flow chart (Fig. 1).

**Table 1.** Baseline characteristics of the clinical variables of 305 patients with COVID-19

Total (*n* = 305)	Survivor (*n* = 245)	Non-survivor (*n* = 60)
Sex		
Men	109 (44.5%)	40 (66.7%)
Women	136 (55.5%)	20 (33.3%)
Fever		
Yes	191 (78.0%)	47 (78.3%)
No	54 (22.0%)	13 (21.7%)
Dizziness		
Yes	14 (5.7%)	3 (5.0%)
No	231 (94.3%)	57 (95.0%)
Fatigue		
Yes	117 (47.8%)	34 (56.7%)
No	128 (52.2%)	26 (43.3%)
Nausea		
Yes	24 (9.8%)	4 (6.7%)
No	221 (90.2%)	56 (93.3%)
Diarrhea		
Yes	43 (17.6%)	12 (20.0%)
No	202 (82.4%)	48 (80.0%)
Muscle pain		
Yes	49 (20.0%)	9 (15.0%)
No	196 (80.0%)	51 (85.0%)
Confusion		
Yes	9 (3.7%)	9 (15.0%)
No	236 (96.3%)	51 (85.0%)
Difficulty in breathing		
Yes	135 (55.1%)	41 (68.3%)
No	110 (44.9%)	19 (31.7%)
Cough		
Yes	158 (64.5%)	41 (68.3%)
No	87 (35.5%)	19 (31.7%)
Expectoration		
Yes	73 (29.8%)	24 (40.0%)
No	172 (70.2%)	36 (60.0%)
CT: exudative lesion		
Yes	58 (23.7%)	12 (20.0%)
No	187 (76.3%)	48 (80.0%)
CT: ground glass shadow		
Yes	52 (21.2%)	5 (8.3%)
No	193 (78.8%)	55 (91.7%)
Hypertension		
Yes	41 (16.7%)	16 (26.7%)
No	204 (83.3%)	44 (73.3%)
Accouchement		
Yes	15 (6.1%)	0 (0.0%)
No	230 (93.9%)	60 (100.0%)

Sixteen clinical variables and 17 clinical measurement variables of 292 patients are shown in Supplementary Table 1 and Supplementary Table 2. Thus, they were used to build the mortality prediction model.

In the first step, based on the sample of 292 patients, the list of relative importance (being greater than 1%) generated by Random Forest and XGBoost is given in Table 2.

**Table 2.** Relative importance values by Random Forest and XGBoost

Random Forest result		XGBoost result	
Variable	Relative importance by Random Forest (%)	Variable	Relative importance by XGBoost (%)
LDH	17.69	LDH	23.74
CRP	8.60	Age	12.10
Lymphocyte count	8.06	Neutrophil	7.09
Age	7.70	Aspartate transaminase	6.69
Blood urea nitrogen	6.69	Lymphocyte count	6.25
Aspartate transaminase	5.69	CRP	6.23
Neutrophil	5.37	Blood urea nitrogen	4.88
White blood cell	5.04	White blood cell	4.42
Creatinine	4.70	Normal platelet	3.61
Normal platelet	3.67	Respiratory rate	3.44
Respiratory rate	3.29	Alanine aminotransferase	3.13
Monocytes	3.24	Systolic pressure	3.12
Systolic pressure	3.21	Creatinine	3.07
Total bilirubin	3.02	Heart rate	2.32
Heart rate	2.82	Total bilirubin	2.18
Diastolic pressure	2.69	Monocytes	2.01
Alanine aminotransferase	2.41	Diastolic pressure	1.98
Temperature	1.92	Temperature	1.60

The two lists contained 18 variables, and they were the same except for their relative importance of the variables. The two lists were also confirmed by both machine-learning methods that were shown to have high AUROC values (Random Forest: 0.9756 and XGBoost: 0.9462) in their average cross-validation results.

In the second step, we conducted 500 bootstrapped samples of 292 patients, and calculated *z*-scores of 18 variables in each bootstrapped sample. Most of the 18 variables are not statistically significant. There were only three variables with statistical significance, namely lactate dehydrogenase (LDH) (*z*-score = 12.08), C-reactive protein (CRP) (*z*-score = 7.24) and age (*z*-score = 21.11). The multivariate logistic regression for mortality prediction based on these three variables are given in Table 3.

**Table 3.** Multivariate logistic regression of three selected variables for mortality prediction

Model	Coefficient	Standard deviation error	Confidence Interval (5%)
Constant	−10.5772	1.794	(−14.093, −7.061)
LDH	0.0076	0.0016	(0.0045–0.0106)
CRP	0.0175	0.0060	(0.0060–0.0289)
Age	0.0857	0.0215	(0.0441–0.1280)

**Fig. 2.**
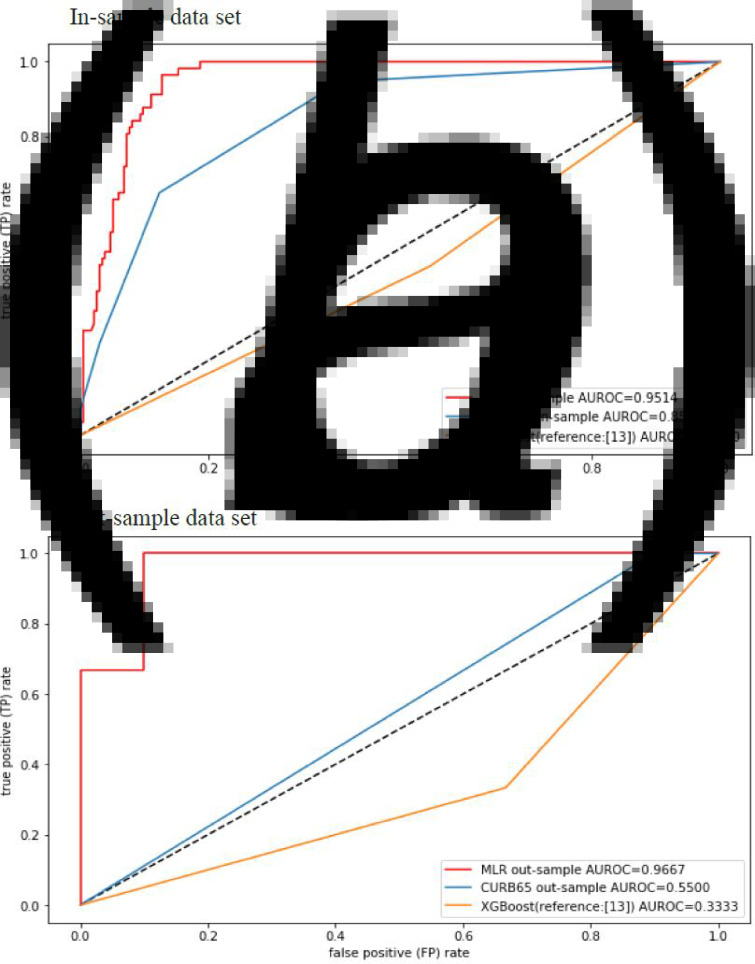
The ROC curves of the obtained mortality model, CURB-65 and the machine-learning-based model on XGBoost13 for different data sets. (a) In-sample data set, (b) out-sample data set.

The ROC curve of the obtained mortality model is given in Figure 2(a). For comparison, the ROC curves of CURB-65 and the machine-learning-based model on XGBoost [13] are also shown in Figure 2(a). It was found that our mortality prediction model (AUROC = 0.9514) was better than both CURB-65 (AUROC = 0.8501) and the machine-learning-based model on XGBoost (AUROC = 0.4530).

The results for the 13 patients out-sample data set with 10 survivor patients (76.9%) and three non-survivor patients (23.1%), the ROC curves of the obtained mortality model, CURB-65 and the machine-learning-based model on XGBoost are given in Figure 2(b). Our mortality prediction model (AUROC = 0.9667) was better than both CURB-65 (AUROC = 0.5500) and the machine-learning-based model (AUROC = 0.3333).

We also compared our mortality prediction model with National Early Warning Score (NEWS) which is a general scheme to check and predict the severity of a patient based on SPO_2_, oxygen inhalation, confusion, heart rate, temperature, respiratory rate and systolic pressure. There were additional measurements of SPO_2_ required, and there were only 200 patients available in the in-sample and out-sample data sets for comparison. It was found that our mortality prediction model (AUROC = 0.9639) was better than NEWS (AUROC = 0.7915) for the combined 200 patients in the in-sample and out-sample data sets.

## Discussion

As for COVID-19, it is crucial to recognise and evaluate the severity of the disease quickly and accurately. This research study was focused on the identification of clinical features of mortality risks in patients with COVID-19 based on clinical data on hospital admission, to find out the key predictive biomarkers which can determine the severity of the disease. From the study we hope to reduce the clinical parameters to be monitored and to use limited medical resources reasonably.

Two laboratory measurement variables (LDH and CRP) provide a reference for predicting a patient's mortality. LDH mainly exists in myocardium, liver, kidney, skeletal muscle or lung and other animal tissues. The increase of LDH reflects, tissue/cell destruction, which is considered as a common symptom of tissue/cell damage [20]. LDH secretion is triggered by cell membrane necrosis, suggesting viral infection or lung injury, such as COVID-19 pneumonia [21]. Studies have shown that tissue damage and inflammation are associated with increased levels of LDH. LDH levels in patients with refractory COVID-19 increased significantly [22]. In addition, when LDH levels are correlated with CT scans, the significant increase in LDH levels reflects the severity of pneumonia [23].

Higher serum CRP can also be used to predict the risk of death in patients with severe COVID-19. CRP is a 21 kD protein, which is mainly synthesised in the liver and found in plasma. It has been considered that the plasma CRP level is an important biomarker to detect the existence of systemic inflammation [24]. Measurement of CRP levels has shown prognostic and/or diagnostic value for many disease states, and in most cases, higher CRP levels are associated with adverse outcomes [25]. In COVID-19, the autopsy showed a large number of sticky secretions from alveolar spillage. It is suggested that COVID-19 may cause inflammatory reactions characterised by deep airway and alveolar injury [26]. Both studies have shown that CRP levels are a powerful indicator reflecting the presence and severity of COVID-19 infection [27, 28].

According to our multivariate regression coefficients (Table 2), it is found that when the levels of LDH or CRP is high, the mortality is high. These results were consistent in the literature [29, 30]. Here we derived the rate of change of mortality with respect to each variable as follows:
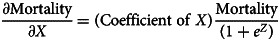
where X = LDH, CRP or Age, and Z = −10.5772 + 0.0076 LDH + 0.00175 CRP + 0.0857 Age

The rate of change of mortality with respect to LDH, CRP or Age is positive as the regression coefficients of LHD, CRP and Age are all positive (see Table 3). It is clear that the mortality increases when LDH, CRP or/and Age increase.

Here simulated examples are studied to understand the rate of change of mortality predicted by our model with respect to the different levels of LDH, CRP and Age. All the values of LDH, CRP and Age here are hypothetical numbers. For example, when Age = 50 and CRP = 80, LDH increases by 1 unit from 500 to 501, the mortality is increased by 0.0014 (from 0.2526 to 0.2512). However, when a patient is younger (Age = 40), the mortality is increased only by 0.0008 (from 0.12464 to 0.12547). The model tells that the rates of change of mortality with respect to LDH can be different for different values of Age. Similarly, when Age = 50 and LDH = 500, CRP increases by 1 unit from 80 to 81, the mortality is increased by 0.0033 (from 0.2512 to 0.2545); when Age = 40, the corresponding mortality is increased by 0.0019 (from 0.1246 to 0.1265). Again, the rates of change of predicted mortality with respect to CRP can be different at different ages. Note that the increase of predicted mortality is 0.0033 with Age = 50 and that of 0.0019 with Age = 40 for 1-unit increase in CRP. While the increase of predicted mortality is 0.0014 with Age = 50 and that of 0.0008 with Age = 40 for 1 unit increase in LDH. We see that the increase of 1 unit in CRP is more dominant in risk than that in LDH.

On the other hand, the increase of the predicted mortality at Age = 50 (0.0014 and 0.0033 for 1 unit increase in LDH and CRP, respectively) is about 1.75 times than the increase of the predicted mortality at Age = 40 (0.0008 and 0.0019) for the 1 unit increase in LDH and CRP, respectively. It is clear that age is the key risk factor in the mortality of a patient which is consistent with the recent study [31]. Previously, it was reported that old age was an important independent predictor of severe acute respiratory syndrome and Middle East respiratory syndrome mortality [4, 32]. This study confirmed that the increase in age is related to the death of individuals with COVID-19.

In this study, we compared our mortality model with the two methods: CURB-65, NEWS and the machine-learning-based model on XGBoost. CURB-65 is required to calculate the score based on confusion, blood urea nitrogen, respiratory rate, systolic pressure/diastolic pressure and age. NEWS is required to calculate the score based on SPO_2_, oxygen inhalation, confusion, heart rate, temperature, respiratory rate and systolic pressure. The machine-learning-based model on XGBoost is required to use LDH, CRP and lymphocyte count. Our ROC curves and AUROC values obtained from our model are better than those by the other three methods. CURB-65 and NEWS are general schemes to predict and check the mortality of a patient. They are not specifically designed for the mortality of a patient with COVID-19.

The machine-learning-based model on XGBoost is a specific scheme to predict the outcome of a patient with COVID-19, i.e., a survivor (a patient is discharged) and a non-survivor (a patient is dead). This machine-learning-based method on XGBoost was trained by using a set of training and testing data set of 404 patients. The data set contains 213 survivors (54.2%) and 191 non-survivors (45.8%). The ratio of non-survivors over the survivors is larger than that in our study and is also larger than the current death rate of COVID-19. This may be the reason why lymphocyte count is included in the XGBoost analysis. In contrast, our machine-learning methods Random Forest and XGBoost did not give the high relative importance in the list of selected variables. Actually, lymphocyte count is not a statistically significant variable in our study. The machine-learning-based method on XGBoost gives a decision tree for predicting the outcome of a patient, while it does not give the mortality risk of a patient directly.

Therefore, our model can be used for early detection of high-risk patients, enabling early intervention. To pave the way for doctors for further prognosing patients with COVID-19. Based on the factors we identified from the study, doctors would be able to launch more appropriate treatment methods on controlling the seriousness of the disease. Our model has the following contributions/advantages. Firstly, the model is simple, concise and easy to use as a guide at hospital admission. Secondly, the model can be adapted to predicting low death rate patients' population's mortality risk which is closer to current death rate of COVID-19. Thirdly, by establishing the association between 24 h of admission and outcome, the model provides a prediction after taking into account the effect of medical treatment during the in-hospital period.

Our model is based on a single-centred study. We also excluded patients with missing values in their medical records. The model can be further improved by implementing our methods to multi-centred, large data-based study.

This study had some limitations. Firstly, our number is relatively small, which limits the possible conclusions. Secondly, the incidence, infection rate and virulence of COVID-19 may be different in different locations and stages of the pandemic, which may limit universality. Thirdly, this study involved only one centre, and the results may not be generalisable to other settings and healthcare systems. Our out-sample data came from the same centre with the in-sample data. Thus, the validity of the comparison result may be questionable. Also, the size of out-sample data is relatively small compared to in-sample, which may reduce the power of model validation too. Fourthly, based on our in-sample data, we could collect more laboratory test results to further enrich our prognosis model. Lastly, compared with the general situation, the percentage of the non-survivors in our data set is still relatively high, we could include more survivors in our study data set to further investigate which factors trigger mortality among COVID-19 patients.

## Conclusion

LDH, CRP and age can be used for identification of severe patients with COVID-19 on hospital admission. We presented a method for predicting mortality risks in patients and providing clinical suggestions for further clinical treatment.

## Data Availability

Data will be available upon request.

## Supplementary material

For supplementary material accompanying this paper visit http://dx.doi.org/10.1017/S0950268820001727. click here to view supplementary material
