# Supplementary material for: Development and validation of prognosis model of mortality risk in patients with COVID-19
Source: Epidemiol Infect. 2020 Aug 4;148:e168. doi: 10.1017/S0950268820001727 (PMC7426607; doi:10.1017/S0950268820001727)
Supplement: Supplementary file 1 [file S0950268820001727sup001.docx]

**Supplementary Table 1** Baseline characteristics of the clinical variables of 292 patients with COVID-19.

| Total  (n=292) | Survivor  (n=235) | Non-survivor  (n=57) | p-value |
| --- | --- | --- | --- |
| **Age** | 53.5 ±14.3 | 68.8 ± 11.4 | 0.000 |
| **Sex**  Men  Women | 103 (43.8%)  132 (56.2%) | 37 (64.9%)  20 (35.1%) | 0.004 |
| **Fever**  Yes  No | 183 (77.9%)  52 (22.1%) | 44 (77.2%)  13 (22.8%) | 0.912 |
| **Dizziness**  Yes  No | 13 (5.5%)  222 (94.5%) | 3 (5.3%)  54 (94.7%) | 0.936 |
| **Fatigue**  Yes  No | 112 (47.7%)  123 (52.3%) | 32 (56.1%)  25 (43.9%) | 0.251 |
| **Nausea**  Yes  No | 23 (9.8%)  212 (90.2%) | 4 (7.0%)  53 (93.0%) | 0.517 |
| **Diarrhea**  Yes  No | 43 (18.3%)  192 (81.7%) | 12 (21.1%)  45 (78.9%) | 0.633 |
| **Muscle pain**  Yes  No | 49 (20.9%)  186 (79.1%) | 7 (12.3%)  50 (87.7%) | 0.140 |
| **Confusion**  Yes  No | 5 (2.1%)  230 (97.9%) | 9 (15.8%)  48 (84.2%) | 0.000 |
| **Difficulty in Breathing**  Yes  No | 128 (54.5%)  107 (45.5%) | 39 (68.4%)  18 (31.6%) | 0.056 |
| **Cough**  Yes  No | 151 (64.3%)  84 (35.7%) | 38 (66.7%)  19 (33.3%) | 0.733 |
| **Expectoration**  Yes  No | 69 (29.4%)  166 (70.6%) | 22 (38.6%)  35 (61.4%) | 0.177 |
| **CT: exudative lesion**  Yes  No | 56 (23.8%)  179 (76.2%) | 11 (19.3%)  46 (80.7%) | 0.465 |
| **CT: ground glass shadow**  Yes  No | 49 (20.9%)  186 (79.1%) | 5 (8.8%)  52 (91.2%) | 0.035 |
| **Hypertension**  Yes  No | 40 (17.0%)  195 (83.0%) | 15 (26.3%)  42 (73.7%) | 0.107 |
| **Accouchement**  Yes  No | 15 (6.4%)  220 (93.6%) | 0 (0%)  57 (100%) | 0.050 |

**Supplementary Table 2** Baseline characteristics of the clinical measurement variables of patients of in-sample data set.

|  | **Survivor** | | | | **Non-survivor** | | | |
| --- | --- | --- | --- | --- | --- | --- | --- | --- |
| Variables | Mean | SD | Min | Max | Mean | SD | Min | Max |
| Alanine aminotransferase | 53.5 | 14.3 | 23 | 93 | 77.2 | 228.5 | 4 | 1747 |
| Aspartate transaminase | 40.1 | 31.0 | 5 | 234 | 93.4 | 299.6 | 9 | 2300 |
| Lactate dehydrogenase | 31.2 | 17.8 | 11 | 136 | 629.3 | 663.4 | 181 | 5193 |
| Normal platelet | 241.6 | 135.6 | 98 | 1188 | 185.9 | 114.8 | 20 | 594 |
| Neutrophil | 221.1 | 83.6 | 23 | 481 | 8.9 | 5.3 | 1.09 | 23.05 |
| Monocytes | 4.6 | 3.2 | 0.73 | 26.24 | 0.4 | 0.3 | 0.05 | 1.3 |
| Total bilirubin | 0.5 | 0.2 | 0.09 | 1.65 | 18.3 | 17.3 | 3 | 123 |
| Temperature | 12.3 | 6.6 | 3.4 | 46 | 37.6 | 1.0 | 36.1 | 40 |
| Heart rate | 37.2 | 1.0 | 35.9 | 42 | 93.9 | 25.4 | 40 | 200 |
| Respiratory rate | 90.9 | 15.0 | 55 | 130 | 26.4 | 7.8 | 16 | 50 |
| Systolic pressure | 22.4 | 13.9 | 2 | 225 | 133.8 | 21.4 | 92 | 190 |
| Diastolic pressure | 128.1 | 17.8 | 75 | 230 | 76.4 | 17.4 | 30 | 142 |
| Creatinine | 81.3 | 12.2 | 50 | 150 | 155.1 | 210.6 | 43.3 | 1040.8 |
| Lymphocyte count | 87.5 | 137.0 | 6.7 | 971 | 0.7 | 0.4 | 0.13 | 2.28 |
| White blood cell | 1.7 | 6.2 | 0.19 | 96.08 | 10.1 | 5.5 | 1.27 | 25 |
| C reactive protein | 6.7 | 8.2 | 1.0 | 123.18 | 80.6 | 50.0 | 0.1 | 183.5 |
| Blood urea nitrogen | 24.4 | 33.9 | 0.1 | 147.4 | 12.9 | 11.4 | 3.36 | 52.83 |

Note: Continuous variables are expressed by mean, standard deviation and the minimum, and the maximum values. Categorical variables are expressed by the number and percentage of patients in each category. There were no missing values in the data set. If the variable is continuous, then the t-test is used to test the statistical significance (p-value) of the difference between the two categories.

**Supplementary Table 3** Variable values of the out-sample data set.

| Total samples  (n=13) | Survivor  (n=10) | | | | Non-survivor  (n=3) | | | |
| --- | --- | --- | --- | --- | --- | --- | --- | --- |
|  | Mean | SD | Min | Max | Mean | SD | Min | Max |
| **Age** | 61.5 | 12.9 | 37.0 | 78.0 | 61.0 | 4.4 | 56.0 | 64.0 |
| **Confusion**  Yes  No | 1 (10.0%)  9 (90.0%) | | | | 0 (0%)  3 (100%) | | | |
| Blood urea nitrogen | 5.6 | 2.1 | 2.0 | 8.9 | 10.4 | 5.7 | 5.4 | 16.6 |
| Respiratory rate | 26.2 | 5.3 | 20.0 | 35.0 | 28.7 | 5.8 | 22.0 | 32.0 |
| Systolic pressure | 136.9 | 16.6 | 110.0 | 161.0 | 122.0 | 25.2 | 101.0 | 150.0 |
| Diastolic pressure | 84.2 | 10.7 | 69.0 | 104.0 | 78.7 | 13.7 | 63.0 | 88.0 |
| Lactate dehydrogenase | 286.4 | 95.0 | 132.0 | 447.0 | 664.0 | 192.2 | 447.0 | 813.0 |
| C reactive protein | 0.8 | 0.4 | 0.3 | 1.5 | 0.7 | 0.2 | 0.5 | 0.8 |
| Lymphocyte count | 52.5 | 38.8 | 4.0 | 106.5 | 121.2 | 37.0 | 81.3 | 154.3 |

**Supplementary Table 4** The machine learning parameters and Multivariate logistic regression parameters setting.

| Machine learning method | Parameters setting |
| --- | --- |
| Random Forest | n_estimators=100, max_depth = 2, max_leaf_nodes = 5 |
| XGboost | n_estimators=100, max_depth = 2, max_leaf_nodes = 3 |
| Multivariate Logistic Regression | random_state=0 |
